# Hypolipidemic effect and activation of Lecithin Cholesterol Acyl Transferase (LCAT) by aqueous extract of *Spirulina platensis* during toxicological investigation

**DOI:** 10.1186/s40795-017-0146-2

**Published:** 2017-03-14

**Authors:** Vicky Jocelyne Ama Moor, Prosper Cabral Nya Biapa, Borgia Legrand Nono Njinkio, Bruno Moukette Moukette, Zacharie Sando, Cyril Kenfack, Baruch Ateba, Marthe Elise Ngo Matip, Constant Anatole Pieme, Jeanne Ngogang

**Affiliations:** 1 Department Physiological Sciences and Biochemistry, Faculty of Medicine and Biomedical Sciences - University of Yaounde 1, Yaounde, Cameroon; 2 Department of Biochemistry, Faculty of Sciences, University of Dschang, Dschang, Cameroon; 3 Department of Pharmarcy and Traditional Medicine, Faculty of Medicine and Biomedical Sciences, University of Yaoundé I, Yaounde, Cameroon; 4 Department of Physics, Cepamoq, University of Douala, Douala, Cameroon; 5 Department of Biochemistry, Faculty of Sciences, University of Ngaoundere, Ngaoundere, Cameroon

**Keywords:** *Spirulina platensis*, Lipid profile, LCAT, HMG CoA reductase, Histopathology

## Abstract

**Background:**

*Spirulina platensis* produced in Nomayos (Cameroon) is used as a dietary supplement. *S. platensis* is known as a neutraceutical with many beneficial effects on humans like lipid-lowering action. This study aims to investigate the mechanism of hypolipidemic action of aqueous extract of *Spirulina platensis* (*S. platensis*) through the toxicological studies.

**Methods:**

In this study, we included two month old Wistar rats, weighing between 180 and 200 g. Aqueous *S. platensis* was extracted and prepared using standard methods. The rats received a supplementation of *S. platensis* at 5000 mg/Kg of body weight as single dose in acute toxicity whereas different doses (250, 500, 1000 mg / kg body weight) were administered in subacute toxicity compared to control. Acute and subacute toxicities were determined according to the guidelines 420 (14 days) and 407 (28 days) of the Organization for Economic Cooperation and Development (OECD) respectively. Biochemical parameters such as urea, creatinine, total and direct bilirubin, lipid profile and transaminases; and histopathological analysis of the liver and kidneys were used to evaluate the toxicity of S. platensis on these Wistar rats. Plasmatic hydroxymethyl glutaryl coenzyme A reductase (HMG CoA reductase) and lecithine cholesterol acyl transferase (LCAT) were performed to explain the lipid-lowering action of *S. platensis*. Histopathological analysis of the liver and kidneys was performed.

**Results:**

Our results show a decrease in total cholesterol for male rats (from 84 to 74 mg/dl) when the dose of *S. platensis* increased; this reduction of the total cholesterol level in male rats was significant at 500 mg/kg. There was also a significant inhibition of HMG CoA reductase in a dose dependent manner between 25 and 84.5 fold compared to the control in both male and female groups. At the dose of 250 mg/kg bw, the level of LCAT was higher compared with other groups and control, but the difference was not statistically significant. A slight inflammation in the liver and the mesangial hyperplasia of the renal glomeruli was revealed by the histopathological investigation in subacute toxicity**.**

**Conclusion:**

*Spirulina platensis* from Cameroon appears to have little toxic effects and may demonstrate hypolipidemic activity through the activation of LCAT.

## Background

Spirulina is a cyanobacteria belonging to the class of Cyanophyceae, Order of Oscillatoriaceae. It is also called Arthrospira, with photosynthetic effects [1]. There are several species of spirulina but the most consumed species by men remains *Spirulina platensis (S. platensis)* also called *Arthrospira platensis. S. platensis* naturally grows in high-salt alkaline water reservoirs in subtropical and tropical areas like America, Mexico, Asian and Central Africa [2, 3]. *S. platensis* is a neutraceutical with many beneficial effects on humans such as antiviral, immunomodulation, immunostimulant, anti-inflammatory, hepatoprotective, anti-cancer, antioxidant and lipid-lowering [4–8].

The hypolipidemic effect of *S.platensis* or its extracts has been demonstrated in various animal models including mouse, rat, hamster and rabbit [9]. The cholesterol lowering activity of *S. platensis* was first reported in albino rats [10], followed by mice [11].

Hyperlipidemia is the presence of high, or abnormal levels of lipids and/or lipoproteins in the blood. There is a correlation between coronary diseases and high levels of lipoprotein [12].

Cardiovascular disease is a major cause of death in developed countries and high cholesterol is an important risk factor in atherosclerosis. Administration of *S. platensis* to volunteer males demonstrated, that although there was no significant increase in high- density lipoprotein (HDL) levels, a significant reduction of Low-density lipoprotein (LDL) cholesterol was noted after 8 weeks [13].

Other research describing the effects of *S. platensis* supplements on patients with ischemic heart disease found a significant reduction in blood cholesterol, triglycerides and LDL cholesterol and an increase in HDL cholesterol [14].

Recently in Cameroon, Ngo-Matip and al showed a reduction of cholesterol and an increased HDL among people living with HIV who consumed *S. platensis* [15]. Nevertheless, since dyslipidemias are a major cause of atherosclerosis, drugs such as statins and even certain herbs are used to lower cholesterol levels. The cholesterol-lowering effect of statins is due to the decrease in the biosynthesis of cholesterol by inhibition of the enzyme 3-hydroxy-3-methylglutaryl coenzyme A reductase (HMG CoA reductase). Some studies have also reported the ex vivo inhibition of HMG CoA reductase activity by herbs such as*, Quercus infectoria, Rosa damascene, Myrtus communis, Andrographis paniculata, Anthocephalus indicus, and Ocimum sanctum* [16, 17].

Recently therapeutic upregulation of lecithine cholesterol acyl transferase (LCAT) function has gained interest as a potential new therapeutic strategy for reducing atherosclerosis. Strategies for therapeutically raising LCAT activity include recombinant LCAT protein administration, viral expression of LCAT, and small molecule activators of LCAT [18].

## Method

### Preparation of aqueous extract of *S. platensis*

*Spirulina platensis* was produced and provided by an NGO named CAP-Spiruline situated at Nomayos (Yaounde- Cameroon).

Aqueous extract of *S. platensis* was obtained by macerating 100 g of *S. platensis* powder with 1000 mL of distilled water. This mixture was then placed on intermittent agitation for 24 h, and the filtrate obtained was immediately lyophilized. The powder obtained was used for various studies. The extraction yield was 16.84% which was calculated using the following formula: Rd = Mass of the extract obtained × 100 / initial powder mass.

### Animal and toxicity material

Before beginning the study, an ethical clearance was obtained (N° 2016/01/699/CE/CNERSH/SP).

*S. platensis* was prepared by dissolving 500 mg of powder in 1 ml of distilled water to obtain a stock solution of 500 mg/ml.

Male and female Wistar rats around two months old and between 180 and 200 g of weigh were used in this study. They were all raised in physiological cage at the Faculty of Medicine and Biomedical Sciences of the University of Yaounde I. They had free access to water and food. The food composition include: carbohydrates (55%), proteins (20%), lipids (3.4%), cellulose (1%), minerals (4.9%), vitamins (1.7%) and moisture (14%). The acute toxicity was determined according to the guideline 420 of the OECD [19]. A total of 20 rats divided into 2 groups of 10 rats for each group (5 males and 5 females) for the control and assay. The rats of the control groups received distilled water while those of the test group received a single dose (5000 mg/kg of body weight) of *S. platensis* dissolved in distilled water. The volume administered at each rat was calculated according to the following formula: Volume (ml) = [dose (mg/kg)* weight (kg)] / concentration (mg/ml).

For sub-acute, the guideline 407 was used [20]. In this study, 40 rats were divided into 4 groups of 10 rats. A group 1 called control received distilled water. The other groups (2–4) received three different doses of *S. platensis* (250, 500 and 1000 mg / kg body weight).

The extract of *S. platensis* was administered through the orally by gavage using a needle once (acute toxicity) and daily up to 28 days (sub-acute toxicity).

After 28 days, the rats were sacrificed after anesthesia with ether; the blood was obtained by cervical dislocation and collected in dry tubes for the determination of biochemical parameters. These parameters included the investigation of urea, total cholesterol, triglycerides, high density lipoprotein cholesterol levels using enzymatic and colorimetric methods (CYPRESS, BELGIUM). The determination of creatinine concentration was carried out by the kinetic Jaffe method (CYPRESS, BELGIUM) while the activities of transaminases (AST and ALT) were performed with kinetic method (CYPRESS BELGIUM).

The activity of hydroxy methyl glutaryl coenzyme A reductase (HMG CoA reductase) was determined in the plasma, by spectrophotometric method as described in the kits (SIGMA ALDRICH, GERMANY). The assay is based on a spectrophotometric measurement of the decrease in absorbance at 340 nm which represents the oxydation of NADPH by the catalytic subunit of HMG CoA reductase in the presence of the substrate HMG CoA. The results are expressed as UI/mg of protein.

The lecithine cholesterol acyl transferase (LCAT) activity was determined by a fluorimetric method using a kit (SIGMA ALDRICH, GERMANY). The fluorescence emission titration were recorded using an optic fiber spectrometer Avantes 2048 (Avantes, Netherlands), with spectral sensitivity within 250–1100 nm range. The LCAT Activity Assay Kit is a fluorometric assay useful for measuring the phospholipase activity of LCAT. We have to mix 1 mL of LCAT Substrate Reagent with 200 mL of Assay Buffer and LCAT source (3–5 mL of plasma or serum). Then to incubate for 4–8 h at 37 °C. After what add 100 mL of the incubated mixture to 300 mL of Read Reagent and measure the fluorescent label (ʎex = 340/ʎem = 390 and 470 nm) and determine the plasmatic ratio (ʎem 470/ʎem 390).

### Histopathology study

The sections of the liver and kidney were fixed in 10% buffered formalin, dehydrated in ethanol, cleared in xylene, and embedded in paraffin. Fixed tissues were sectioned using a microtome. Then, these sections were stained with hematoxylin and eosin and were examined under the optic microscope for toxicant induced changes.

### Statistical analysis

Results are expressed as Mean ± SD of triplicate assays. The factor has been tested using *Kruskal wallis* test and *Dunnett’s* and *Bonforroni* multiple test helped for establishing difference between means. Ponderal growth as well as LCAT activity between male and female at each concentration were analyzed through independent sample *T-test*. The relationship between parameters was achieved with the Spearman rho Correlation Analysis. The differences were considered as significant at *P* < 0.05. SPSS hardware, version 18.0 for Windows helped in analyzing data.

## Results

Physical observations indicated no signs of changes in the skin, fur, eyes mucous membrane, behaviour patterns, tremors, salivation, and diarrhoea of the rats. There was neither mortality observed at the tested dose nor weight loss in the rats receiving *S. platensis*. The fifty percent lethal dose (LD_50_) of *S. Platensis* extract was therefore estimated to be more than 5000 mg/kg. We noted a significant (*p* <0.05) weight gain in male rats ingesting S. *platensis* compared to the control male group (Table 1). This difference was not significant in the group of female rats.

**Table 1:** Weight of the groups of rats

Days	Weight of rats (Mean ± SD)			
	Control female	Test female	Control male	Test Male
Day 1	117,60 ± 10,784	118,6 ± 16,622	117 ± 14,832	117,8 ± 10,426
Day 7	138,40 ± 11,194	146,00 ± 20,261	149,20 ± 16,814	161,60 ± 9,503
Day 14	144,00 ± 8,031	155,60 ± 19,476	167,20 ± 17,669	193,20 ± 13,424
Mean weight gained between day 1 and day 14	26.4 ± 15.59	37 ± 28.50	50.2 ± 22.12	75.4 ± 20.25
*p* value	*p* > 0.05		*p* <0.05	

The Table 2 shows the results of lipid profile during subacute toxicity. The level of total cholesterol decreased in the male groups of rats treated with *S. platensis* compare with control; this reduction was only statistically significant at 500 mg/kg. Regarding the level of HDL cholesterol, only the female group exhibited a significant decrease of concentration mainly at 1000 mg/kg.

**Table 2:** Lipid profile of female and male rats after 28 days of administration of *S. platensis*

Females					Males			
Doses mg/kg bw	TC (mg/dl)	HDL C (mg/dl)	TG (mg/dl)	Atherogenic index CT/HDL C	TC (mg/dl)	HDL C(mg/dl)	TG (mg/dl)	Atherogenic index CT/HDL C
Control	66 ± 23	42 ± 7^a^	250 ± 118	1.64 ± 0.42	92 ± 17^a^	52 ± 6	385 ± 90	1.76 ± 0.14
250	62 ± 12	43 ± 8^a^	257 ± 98	1.44 ± 0.19	84 ± 9^a^	53 ± 9	294 ± 147	1.58 ± 0.17
500	61 ± 11	40 ± 8^a^	207 ± 160	1.50 ± 0.10	74 ± 6^b^	40 ± 3	229 ± 152	1.85 ± 0.32
1000	57 ± 4	35 ± 5^b^	289 ± 127	1.62 ± 0.19	89 ± 14^a^	49 ± 3	334 ± 86	1.81 ± 0.19

In the same column, the values subscripted with the same letter are not significantly different at *p* < 0.05

*Bw* body weight, *TC* total cholesterol, *HDL C* High Density Lipoprotein cholesterol, *LDL C* Low Density Lipoprotein cholesterol, *TG* Triglycerides

The effects of subacute administration of *S. platensis* extract on liver and kidney biochemical parameters are presented in Table 3. Most of these biochemical assays in treated rats were not significantly different from the controls, with the exception of albumin which was low at 500 mg/kg.

**Table 3:** Biochemical parameters of females and males rats after 28 days administration of *Spirulina platensis*

	DOSES mg/kg bw	Alb (g/l)	Urea (g/l)	Creatinine (mg/l)	AST (UI/l)	ALT (UI/l)	T Bili (mg/l)	D Bili mg/l
Females	Control	34.75 ± 1.29	0.59 ± 0.06	9.94 ± 1.33	295.93 ± 32.85	64.11 ± 7.23	1.23 ± 0.48	0.43 ± 0.31
	250	35.65 ± 1.41	0.53 ± 0.05	9.04 ± 0.57	324.05 ± 78.35	71.71 ± 20.47	1.34 ± 0.36	0.39 ± 0.19
	500	33.9 33.98 ± 2.63	0.62 ± 0.12	8.65 ± 1.59	288.70 ± 58.60	68.54 ± 23.08	0.95 ± 0.22	0.32 ± 0.14
	1000	35.01 ± 0.75	0.53 ± 0.10	9.96 ± 1.08	379.43 ± 126.9	77.17 ± 19.96	1.48 ± 0.26	0.55 ± 0.07
Males	Control	34.23 ± 0.94^a^	0.62 ± 0.24	8.97 ± 1.75	344.75 ± 136.46	91.58 ± 37.29	1.07 ± 0.20	0.42 ± 0.19
	250	36.31 ± 0.82^ab^	0.56 ± 0.21	9.30 ± 0.76	337.78 ± 125.44	80.32 ± 18.59	1.26 ± 0.38	0.52 ± 0.42
	500	31.07 ± 2.42^c^	0.62 ± 0.14	9.24 ± 0.67	291.90 ± 22.72	112.39 ± 33.07	1.14 ± 0.41	0.51 ± 0.19
	1000	34.58 ± 0.62^a^	0.61 ± 0.07	9.02 ± 0.33	357.86 ± 75.02	102.29 ± 16.56	1.25 ± 0.26	0.46 ± 0.15

In the same column, the values subscripted with the same letter are not significantly different at *p* < 0.05

*Bw* body weight, *AST* Aspartate Amino transferase, *ALT* Alanine Amino Transferase, *T Bili* total bilirubin, *D Bili* direct bilirubin, *Alb* albumin

Table 4 shows the variations of HMG CoA reductase activity in both groups of rats. In both male and female rats, we found inhibition of the enzyme in rats fed by *S. platensis;* this inhibition is dose-dependent, marked with a dose of 1000 mg/kg. The difference between the groups is statistically significant (*p* < 0.05).

**Table 4:** Activity of HMG CoA reductase of males and females rats after 28 days administration of *Spirulina platensis*

Groups	HMG CoA reductase activity (UI/mg protein)	
	Rats Males	Rats Females
control	0.38 ± 0.001^a^	13.45 ± 0.002^a^
250 mg/kg	0.13 ± 0.002^b^	3.99 ± 0.000^b^
500 mg/kg	0.06 ± 0.000^c^	5.07 ± 0.007^c^
1000 mg/kg	0.02 ± 0.000^d^	0.50 ± 0.000^d^

In the same column, the values subscripted with the same letter are not significantly different at *p* < 0.05

**Fig. 1:**
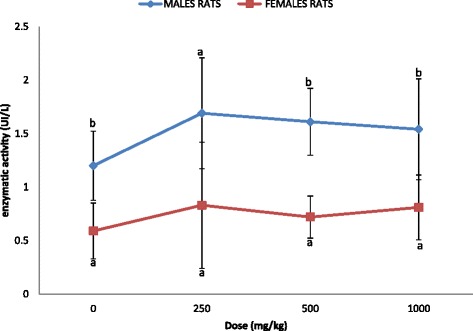
Variation of LCAT activity of male and female rats after 28 days administration of *Spirulina platensis*. The values subscripted with the same letter are not significantly different at *p* < 0.05

The variation in LCAT activity of the rats presented in the Fig. 1 shows that at the dose of 250 mg/kg bw, the level of LCAT was higher both in male and female groups compared to control. However, this variation was not statistically significant within the groups (*p* = 0.399 for males groups and *p* = 0.801 for females groups). When comparing the sex, the level of LCAT was significantly higher in males compared to female except at 250 mg/kg [*p* = 0.02 (control); *p* = 0.07 (250 mg/kg body weight); *p* = 0.003 (500 mg/kg body weight); *p* = 0.04 (1000 mg/kg body weight)].

**Fig. 2:**
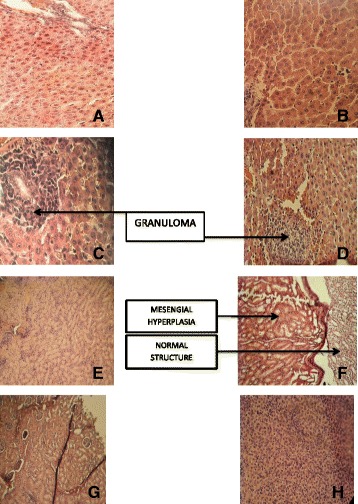
Histopathology of the liver; hematoxylin and eosin X40. (**a**) control (**b**) 250 mg/kg bw (**c**) 500 mg/kg bw (**d**) 1000 mg/kg bw. (**b**) Histopathology of the kidney; hematoxylin and eosin X10. (**e**) control (**f**) 250 mg/kg bw (**g**) 500 mg/kg bw (**h**) 1000 mg/kg bw

Figure 2 shows the histopathological studies of livers and kidneys of the rats fed with *S. platensis*. We found the presence of an inflammatory granuloma especially at the doses 500 or 1000 mg/kg bw. This inflammation is the evidence of lymphocyte multiplication and is not accompanied by cytolysis. The kidney sections show chronic interstitial inflammatory infiltrate microfoci predominant around the tubules with mesangial hyperplasia especially at 250 mg / kg the female rats in particularly. However, at the highest dose, the histopathology of the kidneys is normal.

## Discussion

Various herbs have been used as treatment and prevention for several chronic diseases such as diabetes, hypercholesterolemia and triglyceridemia. In screening natural products for the pharmacological activity, assessment and evaluation of the toxic characteristics of a natural product extract, fraction, or compound are usually an initial step. In our study we investigated the effects of aqueous extract of *S. platensis* on lipid profile mainly on the plasmatic activity of LCAT and HMG-CoA reductase after 28 days of toxicological studies.

Acute toxicity is defined as adverse effects occurring because of short-term administration of a single dose or multiple doses given within 24 h [21]. Acute toxicity tests give a quantitative estimation of acute toxicity, identify target organs and other clinical manifestations of acute toxicity and provide guidance for dose-ranging studies. This test is usually a valid predictor of the response seen in humans [18]. Indeed, the literature reports that the substances with LD_*50*_ higher than 5 g/kg b.w., by oral route are regarded as being safe or practically non-toxic [22]. This is an indication that the aqueous solution of *S. platensis* has negligible level of toxicity when administered orally. At a dose of 5000 mg/kg, S*. platensis* showed no deaths of rats after acute toxicity. Also, the LD (lethal dose) value for phycocyanin a main active component of S. platensis extract has been higher than 5000 mg/kg [23].

Furthermore, the diets and water were well-accepted by the rats treated with *S. platensis* extract suggesting the extract did not cause any alterations in carbohydrate, protein or fat metabolism in these experimental animals. Our results also showed that the *S. platensis* extract did not adversely interfere with the nutritional benefits, such as weight gain and stability of the appetite. The body weight changes serve as a sensitive indication of the general health status of animals [24].

Subchronic toxicity studies on *S. platensis* have been conducted by some researchers [21]. Our toxicological study brings some differences, mainly in the feeds given to the animals, the time of administration, and the kind of analysis conducted. The exploration of liver and kidney function is very important in the investigation of toxicity of drugs and plant extracts as they are both necessary for the survival of an organism [25]. High levels of ALT, AST, and alkaline phosphatase (ALP) are reported in liver diseases or hepatotoxicity [26]. No significant changes was found in ALT, AST, in our study in both male and female rats at all doses suggesting that the administration of *S. platensis* extract does not affect the hepatocyte function in the rats. In a chemopreventive study of *S. platensis* against deltamethrin, Abdel-Daim and al [27] observed a reduction of hepatic markers (transaminases, alkaline phosphatase, bilirubin, total cholesterol) and renal markers (urea, creatinine, uric acid). The liver sections revealed significant lymphocytic multiplication. Previous studies showed the hepatoprotective effects of *S. platensis* and its active constituents against drugs, chemicals and xenobiotics [28–30].

The protective role of *S. platensis* could be attributed to the presence of ß-carotene, superoxide dismutase, selenium and blue pigment phycocyanin in the algae [5]. Phycocyanin significantly reduced the hepatotoxicity caused by paracetamol which induces the formation of free radicals. The hepatoprotective effect of phycocyanin was therefore attributed to the inhibition of reaction involved in the formation of reactive metabolites and possibly due to its radical scavenging activity. Also, a study has revealed that the hepatoprotective mechanism of *S. platensis* passes through the presence of phycocyanin and is related to the blockage of inflammatory infiltration by inhibiting the expression of tumor growth factor-beta 1 and hepatocyte growth factor [31, 32].

Our study demonstrated no changes in plasma urea and creatinine levels in *S. platensis* extract treated groups indicating a normal renal function. The nephroprotective effects of *S. platensis* have been reported against renal injury induced by gentamicin [33] as well as oxalate [34]. The effective compound responsible for the suppression of renal toxicity seems to be phycocyanin [35].

Changes in the parameters of the lipid profile between different groups of rats were not significant, except for cholesterol.

Cholesterol reduction and related effects of *S. platensis* have been reported mostly in subjects with elevated blood lipid levels [36]. It has been shown that administration of *Arthrospira maxima* (specie of Spirulina) associated with simvastatin prevents the acute fatty liver induced by the administration of ethanol and a hypercholesterolemic diet to mice [37].

The hypocholesterolemic effects of *Arthrospira maxima* are in accordance with other reports on rats when it was given in a normal diet [38] with a high-fructose diet [39] or when *Arthrospira maxima* was administered to humans, 4.2 g / day during 4 weeks [13]. Hyperlipidemia lowering effect of *S. platensis* seems to be more sensitive in an experimental model than in a normal blood lipid range [40].

The cholesterol homeostasis is very important for the human health. HMG COA reductase is the rate-limiting step in the biosynthesis of cholesterol in humans: it converts HMG COA into mevalonate. The inhibition of this enzyme would be an effective means of lowering plasma cholesterol. It has been suggested that exogenous cholesterol inhibits the expression of mRNA for of 3-hydroxy-3-methyl coenzyme A reductase (HMG CoA reductase) [39, 41] that appears after the intracellular mevalonate deficit in the liver. The inhibition of HMG CoA reductase produces a depletion of the mevalonate metabolites which is critical for cellular viability [24, 42]. Regulation of HMG CoA reductase activity is the primary approach for controlling de novo cholesterol synthesis, while abnormal activation can lead to hepatic cholesterol accumulation and hypercholesterolemia. Full suppression of the reductase requires the presence of at least two regulators: cholesterol, which is normally derived exogenously from plasma low density lipoprotein (LDL), and a non-sterol product, which is normally synthesized endogenously from mevalonate [16]. The major metabolic pathway for reducing cholesterol is via conversion to bile acids or preventing the cholesterol synthesis by inhibiting the HMG CoA reductase enzyme. In our study, we found that the decrease of plasmatic HMG COA reductase in the plasma of the treated group was between 2.9 and 26.9 fold higher compared to control. Moreover, the ratio between HMG CoA reductase of the female and male rats varied from 25 to 84.5 with the maximum at the dose of 250 mg/kg bw. These results confirmed that the administration of *S. platensis* inhibits the plasmatic activity of HMG COA reductase significantly in the male than the female rats. The enzyme inhibitory effect correlates with the medicinal properties and phytochemical components of *S. platensis* such as phenol and flavonoids compounds. Some studies have also reported the ex vivo inhibition of HMG CoA reductase activity by herbs specially medicinal plants, namely*, Quercus infectoria, Rosa damascene, Myrtus communis, Andrographis paniculata, Anthocephalus indicus, and Ocimum sanctum* [16, 17]. Clinical trials have shown that the use of HMG CoA reductase inhibitors in patients with coronary risk improved endothelial function through reduction of oxidative stress and/or up regulation of NO activity [43].

Lecithin cholesterol acyltransferase (LCAT) is one of the major modulators of plasma high-density lipoprotein cholesterol (HDL-C) and plays a central role in the reverse cholesterol transport (RCT) process [44]. LCAT is a plasma enzyme that circulates mostly in association with the high density lipoproteins (HDL) and is responsible for the synthesis of cholesterol esters present in human plasma. Cholesterol esterification catalyzed by LCAT also reduces the amount of unesterified cholesterol in plasma. The level the highest level of LCAT was noted at 250 mg/kg bw demonstrating the increase of esterification of cholesterol which lead to the synthesis of HDL-C. These findings corroborate with the higher concentration of HDL-C noted in the group of rats receiving 250 mg/kg of *S. platensis* extract. Therefore LCAT plays a key role in the incorporation of free cholesterol into HDL and its transfer back to VLDL and LDL, which are later returned in liver cells [45]. Our study has shown that the synergistic effect of HMG CoA reductase inhibition and LCAT activation reduced the level of cholesterol in the group of rats treated with *S. platensis.*

## Conclusions

Administration of aqueous extract of *S. platensis* from Nomayos in Cameroon resulted in little toxic effects. It demonstrates hypolipidemic activity through an activation of LCAT. The findings provide preliminary data that suggest that *S. platensis* is capable of reducing total cholesterol. Further studies are needed to confirm this result by inducing hypercholesteromia.

## Abbreviations

Dw: Dry weight
HDL: High Density Lipoproteins
HIV: Human Immunodeficiency Virus
HMG CoA reductase: Hydroxy Methyl Glutaryl Coenzyme A Reductase
LCAT: Lecithine Cholesterol Acyltranferase
LD: lethal dose
LDL: Low Density Lipoproteins
NGO: Non-Governmental Organization
OECD: Organization for Economic Cooperation and Development
